# Current Trends in MOF (Metal-Organic Framework) and Metal X-ides

**DOI:** 10.3390/ijms241311188

**Published:** 2023-07-07

**Authors:** Xinxin Wen, Lili Lin, Siwei Li

**Affiliations:** 1 Institute of Industrial Catalysis, School of Chemical Engineering and Technology, Xi’an Jiaotong University, Xi’an 710049, China; xinxinxin@stu.xjtu.edu.cn; 2 Institute of Industrial Catalysis, State Key Laboratory of Green Chemistry Synthesis Technology, College of Chemical Engineering, Zhejiang University of Technology, Hangzhou 310014, China

Metal–organic frameworks (MOFs) are a class of porous two- or three-dimensional infinite structure materials consisting of metal ions or clusters and organic linkers, which are connected via coordination bonds [1]. Owing to their extraordinarily high surface area, tunable pores, and diverse functional sites, MOFs have become one of the most widely investigated materials for wide applications in gas separation [2], catalysis [3], drug delivery [4], optical and electronic applications [5], and sensing [6]. Moreover, MOFs also play a significant role in nanomaterial synthesis. By applying different thermal and/or chemical treatments, MOFs can be used as sacrificial templates and precursors for oxides, hydroxides, sulfides, phosphides, nitrides, and carbides (i.e., X-ides) [7,8]. The aim of this Special Issue is to summarize the application of MOFs and X-ides in catalysis, environmental biosystems, and electromagnetic (EM) adsorption.

Wang et al. [9] prepared a series of dual-shell Mo_2_C/C nanospheres for microwave absorption. The dual-shell structure could optimize impedance matching by prompting the intrinsic impedance to be as close as possible to that of the outside air. It was proven that the dual-shell structure of the Mo_2_C nanoparticles had a positive effect on EM energy attenuation. This finding could facilitate the design and preparation of highly efficient carbon-based microwave-absorbable materials.

Li et al. [10] synthesized a Zn_3_In_2_S_6_/g-C_3_N_4_ photocatalyst by using a low-temperature solvothermal method, showing excellent degradation performance regarding tetracycline (TC) under visible light irradiation. The degradation mechanism of photocatalysts on TC was analyzed, demonstrating excellent performance at low temperatures. This study could provide a new strategy for the preparation of photocatalysts. 

Xia et al. [11] developed a strategy to facilitate photocatalytic reactions by designing a composite architecture of ZIF–8 ligands binding to ZnO seed layers on carbon fibers during in situ synthesis, which exhibited superior photodegradation performance with respect to TC. This recent study provides useful insights into the design of other photocatalysts for the treatment of wastewater.

Lin et al. [12] recently summarized the progress of MOF-based CO_2_ hydrogenation catalysts and compared their synthetic strategies, unique features, and enhancement mechanisms with traditionally supported catalysts. The challenges and opportunities pertaining to the precise design, synthesis, and applications of MOF-based CO_2_ hydrogenation catalysts were also summarized. Great emphasis was placed on the various confinement effects involved in CO_2_ hydrogenation. The mechanistic insights into the confinement effects associated with MOFs or MOF-derived catalysts designed for CO_2_ hydrogenation provided by this review could facilitate the development of clear structure–activity relationships, aiding rational catalyst design.

Wang et al. [13] reviewed the recent progress of MOFs and MOF-derived materials, as well as their function and influences on performance in environmental biosystems. In this review, the interaction between these biocomponents and MOFs (or MOF-derived materials) was also discussed, also helping to guide the rational design of MOF-related materials and facilitate a better understanding of their interaction with biocomponents.

Xu et al. [14] summarized the role and development of the d-band center of materials based on the iron-series metals used in electrocatalytic water splitting. This review mainly focused on the influence of changes in the d-band centers of different iron-based material composites on the performance of electrocatalysis. Such information could be helpful for adjusting the active centers of catalysts and improving electrochemical efficiency in future studies.

Ju et al. [15] described the state-of-the-art applications of metal and metal oxide nanomaterials in the treatment of bacterial infective diseases. This progress report provided new insights into the mechanisms of action of metal and metal oxide nanomaterials in antibacterial and antibiofilm applications and could further facilitate their development.

Zhao et al. [16] reviewed the progress of research on the synthesis and application of MIL-101(Cr). This review focused on the applications of MIL-101(Cr) and its associated synthesis strategies, with special emphasis being placed on the on the fields of adsorption and catalysis. This review could promote further developments in these fields.
